# Supplementary material for: The chemokine receptor CCR8 is not a high-affinity receptor for the human chemokine CCL18
Source: PLoS One. 2024 Sep 11;19(9):e0305312. doi: 10.1371/journal.pone.0305312 (PMC11389940; doi:10.1371/journal.pone.0305312)
Supplement: Attachment — Submitted filename: Response to Reviewers 2024 08 08 MJS.docx [file pone.0305312.s001.docx]

**Hussain et al. “The Chemokine Receptor CCR8 is Not a High-affinity Receptor for the Human Chemokine CCL18”**

**Response to Reviewers**

**Reviewer 1**

“Negative” results are never easy to publish in peer-reviewed journals and this certainly applies to the present study by Hussain and co-workers. Human CCL18 has been reported to be a second, in addition to CCL1, chemokine ligand for human CCR8, based on in vitro data showing chemotactic activity and direct interaction with CCR8. Furthermore, a recent report in Cell Rep. Med. by the same group provides evidence for in vivo activity of CCL18 for T cells in a human skin transplant model. Interestingly, Figure 4i/j in the original report even demonstrated that human CCL18 is a chemokine ligand for murine CCRR8. Now here, the present study using the same cellular systems (and much more) does not corroborate the initial findings. The study was carefully carried out by multiple laboratories yet did not find any evidence for a high-affinity interaction of CCL18 (and N-terminal truncation variants) with CCR8 or an induction of prototypic cellular responses, including chemotaxis and CCR8 signaling events. These results suggest that CCL18 is at best a low-affinity ligand for CCR8. Considering the importance of CCR8 in pathologies, it is essential that the present findings are made public in the hope that it may instigate future discussions leading to the clarification of this conundrum.

The authors are invited to address the following points.

1) Figure 3 shows that CCL18 was unable to trigger CCR8 internalization. CCL1 was used as a positive control. Are you sure that CCL1 led to CCR8 internalization as opposed to inhibition of Ab 433H binding?

Response: This is a good point and something that we did assess by including a matched incubation condition in which CCR8 transfectants were incubated with CCL1 at 4 °C (to inhibit endocytosis) for the same 30-minute time period and processed identically to those treated at 37 °C (allowing endocytosis). This is shown here as the raw median fluorescence values from six experiments which we have included as an additional panel in Figure 3 (Figure 3C). It can be seen that much of the loss of 433H binding is attributable to CCL1 binding to CCR8, although there is even further reduction in 433H binding when the cells are incubated at 37 °C, suggestive that CCL1 does lead to CCR8 internalisation. Either way, neither CCL18 nor CCL17 is able to significantly inhibit 433H binding to CCR8 suggesting that they do not productively interact with the receptor.

2) CCR8 signaling induces strong Ca^2+^ mobilization responses. Is there any reason why this assay was not included in the study?

Response: Neither the Monash University group nor the Imperial College London group has a suitable instrument to obtain high-quality Ca^2+^ mobilisation data. While it would have been ideal to obtain such data, we consider the assays described in the manuscript to be sufficient to demonstrate lack of CCR8 activation by CCL18.

3) In Figure 7, the study was extended to include murine CCR8 and the newly discovered ligand murine CCL8. I am not certain how these data help to resolve the human CCL18 questions. Perhaps it would be best to more carefully examine the murine chemokine system in a separate study?

Response: We included these data simply because the Luster lab had reported mCCL8 to be a “functional analogue” of human CCL18. We agree that a separate study is needed to further examine the properties of mCCL8, particularly whether it is an agonist for other murine receptors. While we don’t have the resources to pursue this in detail, the preprint of this manuscript has already prompted discussions with the Luster lab and they are keen to pursue such a study. We will remain in contact with them and share any relevant data we have.

4) Overall, the “negative” data are convincing, and it is puzzling that the results obtained with CCR8-transfected 4DE4 cell lines by the two groups gave such conflicting results. The striking discrepancies beg for a scientific explanation?

Response: We totally agree and wish we had a convincing explanation. Our manuscript explores several possible explanations, including differences in cell culture conditions and differences in the N-terminus or C-terminus of the CCL18 variant used. Unfortunately, we were unable to find evidence supporting these hypotheses. While it is possible that mistakes were made in the previous study, we are reluctant to make such a speculation in print as we have no evidence for this. While we have taken this study as far as we can, we agree with the reviewer that it is important our results should be published to stimulate and facilitate further investigations.

**Reviewer 2**

In their manuscript Hussain et al describe a detailed evaluation of the potential (previously reported) interaction between human CCL18 and the chemokine receptor CCR8 as well as the potential interaction of this receptor with murine CCL8. CCL18 is a highly abundant chemokine in blood and upregulated in multiple pathologies. As such, it is crucial that we understand its interaction with potential chemokine receptors. Authors convincingly show using multiple cell lines that human CCL18 and murine CCL8 do not signal through the human CCR8. The experiments have been carefully performed, confirmed in multiple assays and on multiple cell lines using a broad concentration range of the potential ligand. In addition, since CCL18 might be processed by proteases, authors also investigated whether proteolytic processing might activate CCL18 on CCR8. However, also the processed CCL18 forms (as well as murine CCL8) failed to signal through CCR8.

No response necessary.

**Editor Comments**

Both reviewers appreciate your manuscript, however, at least one reviewer and myself found a few points which should be clarified. Therefore, we invite you to submit a revised version of the manuscript that addresses the points raised during the review process.

- Reference 27 should be edited as the manuscript cannot be found

Response: Thanks for pointing this out. This is a thesis from Imperial College London. We have edited the reference to include the open access URL (<http://hdl.handle.net/10044/1/78542>).

- Currently three receptors for CCL18 are reported in the literature GPER (DOI: 10.1002/jcp.22284, 10.21873/anticanres.14291), CCR6 (10.3389/fimmu.2024.1327051; doi:10.3390/cells13030238), and PITPNM3 (doi: 10.1016/j.ccr.2011.02.006). Nevertheless. I agree with your doubts on PITPNM3.

Response: We were not previously aware of the report that GPER (aka GPR30) is a receptor for CCL18. The initial report of this by Catusse et al. (2010) showed that CCL18 did not directly induce Ca^2+^ flux or chemotaxis but, instead, CCL18 suppressed these responses to CXCL12. The evidence demonstrated fairly convincingly that this effect of CCL18 was mediated by the receptor GPR30. However, these observations do not account for the ability of CCL18 to stimulate signalling and chemotaxis in T cells. The later paper by Schmidt-Wolf et al. (2020) sought to explore the signalling of GPR30 in response to CCL18 but none of the reported data for CCL18 were statistically significant. We have now cited the Catusse paper in the Introduction (page 4) and both papers in the Discussion (page 15).

Regarding CCR6, two very recent (2024) papers have presented evidence that CCR6 mediates the CCL18-stimulated migration of mouse and human T cells as well as a pro-fibrotic phenotype of fibroblasts. Although these reports were too recent for us to have considered them during our study, they now suggest that CCR6 may be the elusive CCL18 receptor. We have now noted these recent findings and cited the two papers (refs 45 and 46) in the Discussion (page 15, marked up manuscript).

Reviewer 1 raised some additional questions which should be answered.

Response: These are addressed above.

**Additional Edit**

We have removed a reference to an accompanying paper (bottom of page 14, tracked version) as that manuscript is still under review so will not be published back-to-back with the current manuscript.
